# Chloroquine resistance is associated to multi-copy *pvcrt*-*o* gene in *Plasmodium vivax* malaria in the Brazilian Amazon

**DOI:** 10.1186/s12936-018-2411-5

**Published:** 2018-07-16

**Authors:** Siuhelem Rocha Silva, Anne Cristine Gomes Almeida, George Allan Villarouco da Silva, Rajendranath Ramasawmy, Stefanie Costa Pinto Lopes, André Machado Siqueira, Gabriel Luíz Costa, Taís Nóbrega Sousa, José Luiz Fernandes Vieira, Marcus Vinícius Guimarães Lacerda, Wuelton Marcelo Monteiro, Gisely Cardoso de Melo

**Affiliations:** 1 Fundação de Medicina Tropical Dr. Heitor Vieira Dourado, Manaus, Amazonas 69040-000 Brazil; 2 Universidade do Estado do Amazonas (UEA), Manaus, Amazonas 69040-000 Brazil; 3 Instituto Nacional de Infectologia, Evandro Chagas, Fiocruz, Rio de Janeiro, 21040-360 Brazil; 4 Centro de Pesquisas René Rachou, Fiocruz, Belo Horizonte, Minas Gerais 30190-002 Brazil; 5 Instituto Leônidas & Maria Deane (ILMD), Fiocruz, Manaus, Amazonas 69057-070 Brazil; 6 Universidade Federal do Pará, Belém, Pará 66010-010 Brazil

## Abstract

**Background:**

The resistance of *Plasmodium vivax* to chloroquine has become an obstacle to control strategies based on the use of anti-malarials. The current study investigated the association between *P. vivax* CQ-resistance in vivo with copy number variation and mutations in the promoter region in *pvcrt*-*o* and *pvmdr1* genes.

**Methods:**

The study included patients with *P. vivax* that received supervised treatment with chloroquine and primaquine. Recurrences were actively recorded during this period.

**Results:**

Among the 60 patients with *P. vivax*, 25 were CQ-resistant and 35 CQ-susceptible. A frequency of 7.1% of multi-copy *pvcrt*-*o* was observed in CQ-susceptible samples and 7.7% in CQ-resistant at D0 (P > 0.05) and 33.3% in CQ-resistant at DR (P < 0.05). For *pvmdr1*, 10.7% of the CQ-susceptible samples presented multiple copies compared to 11.1% in CQ-resistant at D0 and 0.0% in CQ-resistant at DR (P > 0.05). A deletion of 19 bp was found in 11/23 (47.6%) of the patients with CQ-susceptible *P. vivax* and 3/10 (23.1%) of the samples with in CQRPv at D0. At day DR, 55.5% of the samples with CQRPv had the 19 bp deletion. For the *pvmdr*-*1* gene, was no variation in the analysed gene compared to the *P. vivax* reference Sal-1.

**Conclusions:**

This was the first study with 42-day clinical follow-up to evaluate the variation of the number of copies and polymorphisms in the promoter region of the *pvcrt*-*o* and *pvmdr1* genes in relation to treatment outcomes. Significantly higher frequency of multi-copy *pvcrt*-*o* was found in CQRPv samples at DR compared to CQ-susceptible, indicating parasite selection of this genotype after CQ treatment and its association with CQ-resistance in vivo.

**Electronic supplementary material:**

The online version of this article (10.1186/s12936-018-2411-5) contains supplementary material, which is available to authorized users.

## Background

Malaria remains a major public health problem in many countries. Globally, 3.2 billion people are exposed to its transmission in 91 countries and territories around the world. In 2016, an estimated 216 million cases of malaria occurred worldwide. About 4% of estimated cases globally are caused by *Plasmodium vivax* [1]. In some countries, such as Brazil, the current treatment for *P. vivax* is based on two anti-malarials, chloroquine (CQ) and primaquine (PQ) [2]. One of the greatest threats to the control and elimination of malaria is the spread of parasites resistant to anti-malarial drugs. *Plasmodium vivax* resistance to chloroquine (CQRPv) is difficult to detect due to the low level of parasitaemia among parasite carriers and also to distinguish from relapses to reinfections [3].

The first reports of CQRPv were from 1989 [3, 4], 30 years after the resistance report for *Plasmodium falciparum*. In Brazil, the first reported case of CQRPv was from a patient treated in Manaus, in the Brazilian Amazon [5]. In this city, other subsequent studies assessed the efficiency of standard supervised therapy and the proportion of CQ treatment failures was 10.1% in 2007 [6] and 5.2% in 2014 [7].

*pfcrt*-*o* and *pfmdr1* are molecular markers involved in CQ resistance in *P. falciparum* [8]. Several studies sought mutations in these genes orthologs in *P. vivax* samples and the majority did not show the involvement of these genes with CQ resistance [9, 10], suggesting that in this species the mechanism is probably different. In an in vivo study, in Manaus, *P. vivax* isolates from patients treated with CQ with treatment failure presented an increased *pvcrt*-*o* and *pvmdr*-*1* gene expression on both D0 (day of admission) and DR (day of recrudescence) compared to patients that responded properly to treatment. No significant differences were observed between immature and mature asexual forms in CQRPv and susceptible groups [11]. Other study cited that *pvcrt*-*o* transcription is not a primary determinant of ex vivo drug susceptibility, but is rather related to stage of parasite development [12].

An AAG insertion in the exon I of *pvcrt*-*o* gene leading to an extra amino acid at position 10 (termed K10 insertion) was identified as a possible marker for CQRPv [13]. In Myanmar, more than half of the samples (63.9%) showed the K10 insertion [14]. During later years there have been an increasing number of whole genome sequences for *P. vivax* from Latin America where polymorphisms in potential drug resistance genes are mentioned [15, 16]. These studies showed that the genetic basis of drug resistance can be gained simply by examining the genomes of parasites.

This study aimed to investigate the association between CQRPv with copy number variation and mutations in the promoter region in *pvcrt*-*o* and *pvmdr1*.

## Methods

### Ethics statement

The study was approved by the Ethics Review Board of *Fundação de Medicina Tropical Dr. Heitor Vieira Dourado* (FMT-HVD) (approval number 426/2011). Participants were informed about the study and signed a consent form. In the case of under 18 years, the study participant signed an assent form and the guardians signed a consent form.

### Location of the study

The study was conducted from January 2012 to April 2013 at the FMT-HVD, an infectious disease referral centre located in Manaus, in the Western Brazilian Amazon.

### Selection of patients

The study included patients with *P. vivax* malaria of both genders, aged 6 months–60 years, weight greater than 5 kg, with blood parasite density from 250 to 100,000 parasites/mL and axillary temperature ≥ 37.5 °C or history of fever in the last 48 h. Use of anti-malarials in the previous 30 days, impossibility to be followed up for 42 days and clinical complications were considered exclusion criteria [17]. Patients received supervised treatment with 25 mg/kg of CQ phosphate over a 3-day period (10 mg/kg on days 0 and 7.5 mg/kg on days 1 and 2. PQ was prescribed at the end of follow-up or if malaria recurred during follow-up, at the dosage of 0.5 mg/kg per day, during 7 days.

Patients were evaluated on days 0, 1, 2, 3, 7, 14, 28 and 42 and, if they felt ill, at any time during the follow-up period. Day 0 (D0) is defined as the admission day and day of recurrence (DR) as the day of recrudescence for the same patient. Both samples from the same patient were analysed whenever available. For some patients, only a one-time sample (D0 or DR) was available. Thick blood smears and full blood counts were collected from all patients on each visit. On D0 and in DR, whole blood was also collected for DNA storage. CQ and desethylchloroquine (DCQ) plasmatic levels were determined only in case of parasitological failure. Three aliquots of 100 μL of plasma from DR samples were spotted onto filter paper for later analysis by high performance liquid chromatography to determine the levels of CQ and DCQ, as previously described [2, 18]. CQRPv was defined as a parasite recurrence presenting plasma concentrations of CQ plus DCQ higher than 100 ng/mL when added up. Susceptible-CQ *P. vivax* group consisted of patients with no parasite recurrence during the follow-up period.

### *Plasmodium vivax* diagnosis

Thick blood smear was performed by the Walker technique [19] and evaluated by an experienced microscopist. Parasite densities were calculated by counting the number of parasites per 500 leukocytes and the number of parasites/µL per patient was determined [20]. Real-time PCR was performed to confirm *P. vivax* monoinfection. The extraction of total DNA from whole blood was performed using the QIAamp DNA Blood Mini Kit (Qiagen, USA), according to the manufacturer’s protocol. The DNA was amplified in an Applied Biosystems 7500 Fast System using primers and TaqMan fluorescence labeled probes for real-time PCR [21].

### Copy number variation of *pvcrt*-*o* and *pvmdr*-*1*

Quantitative PCR (qPCR) was performed using Taqman^®^ Universal PCR Master Mix (Applied Biosystems) and 100–200 ng of template DNA were prepared from each sample. PCR products were amplified and identified on a 7500 FAST (Applied Biosystems). Sequences of primers used to determine copy number variation of *pvcrt*-*o* [22] and *pvmdr*-*1* [23] genes were described in Additional file 1.

Cycling parameters for PCR were an initial denaturation step at 95 °C for 10 min, 40 cycles of 15 s at 95 °C and 1 min at 60 °C. The single-copy β-tubulin gene was used as a reference gene (normalizer), and a field sample with a single copy of the target gene was used as a calibrator. The ΔΔCt method was used to determine the number of copies of *pvcrt*-*o* and *pvmdr*-*1* genes relative to a standard calibrator sample. Samples were considered to have a single copy of the gene when the estimated value was 0.5–1.5, while values above 1.5 were defined as gene amplification. Each DNA sample was assayed in triplicate.

To define the calibrators for copy number variation (CNV) analysis using real-time PCR assays, plasmids containing a single copy of each gene: *pvcrt*-*o*, *pvmdr*-*1* and *pvtubulin* were constructed. The presence of a unique copy of each gene was confirmed by sequencing. Using the single-copy plasmids, a set of field samples to define the gene copy number using relative quantification in real-time PCR was evaluated. Thus, samples with a single copy of *pvcrt*-*o* and *pvmdr*-*1* were selected to be used as calibrators in the CNV assays.

### Nucleotide sequencing of the promoter region of *pvcrt*-*o* and *pvmdr*-*1* genes

PCR primers and different reaction conditions used to amplify *pvcrt*-*o* and *pvmdr*-*1* gene sequences are described in Additional file 2. Sequencing reaction was performed in triplicate. Nucleotide sequences were analysed using the NCBI BLAST algorithm and compared with reference sequences of GenBank [24]. Amino acid sequences were aligned and compared with sequences of the reference chloroquine transporter from *P. vivax* Sal1 (GeneBank ID: NC_009906) using Geneious^®^ software (Biomatters, v6.0.5).

### Polymorphism analysis of msp*1*F3, MS2 and MS8 molecular markers for patients with CQRPv

For the genotyping procedures for comparing day 0 and recurrences samples, 3 highly polymorphic genes were chosen according to diversity and discriminatory potential in the region, msp1F3, MS2, and MS8 [25, 26]. PCRs were performed in 20 µL reactions with 10 µM of each primer, 2 µL of 10 × Buffer B (50 mM KCl, 10 mM Tris, pH 8.3), 2.5 mM each dNTP, 25 mM MgCl2, 1.5 U Taq DNA polymerase and 5 µL genomic DNA, as described in Additional file 3. All reagents were used from Invitrogen. 3 µL of diluted primary PCR product was used as template for the nested PCR. PCRs were performed with conditions as follows: initial denaturation at 95 °C for 1 min, followed by 29 cycles (primary PCR) or 24 cycles (nested PCR) of denaturation at 95 °C for 30 s, annealing at 59 °C for 45 s, elongation at 72 °C for 1 min, followed by a final elongation at 72 °C for 5 min. PCR products were sent to Macrogen for capillary electrophoresis-based sequencing and analysed with Gene Marker version 2.6.0. To distinguish reinfection, recrudescence, and relapse by *P. vivax*, was used the classification method recommended by WHO for *P. falciparum* [27]. For each recurrence, samples were classified as homologous if at least 1 allele for each locus investigated was detected in both paired samples (D0 and DR) on two different markers and as heterologous if all alleles for a given marker were different on two or more markers [11].

### Statistical analyses

Data were analysed using SPSS^®^ version 21.0 for Windows (SPSS Inc.^®^ Chicago, IL, USA). Normal distribution of data was evaluated with the Kolmogorov–Smirnov test. Asexual parasitaemia at D0, D1, D2 and D3 was compared in patients with CQRPv and CQ-susceptible *P. vivax* using T-Student test. Parasitaemia clearance rates at D1, D2 and D3 were compared in patients with CQRPv and CQ-susceptible *P. vivax* using Chi square or Fisher’s test. Chi square or Fisher’s test was used to test differences in proportions of polymorphism of the promoter region and copy number variation. A *p* value of 0.05 or lower was considered to be statistically significant. The genetic variation for each microsatellite locus was measured by calculating the expected heterozygosity (H_E_). H_E_ was calculated using D0 and DR for each locus as = [n/(n − 1)] [1 − ∑p_i_^2^] where n is the number of isolates sampled and p_i_ is the frequency of allele i.

## Results

Sixty patients with *P. vivax* were included in the study. Twenty-five patients presented CQRPv and 35 presented CQ-susceptible *P. vivax.* Clinical and laboratory characteristics of the patients with CQRPv and CQ-susceptible *P. vivax* are presented in Tables 1, 2. Of the patients with CQRPv, 80% were male, with a mean age of 24.6 years. The mean haemoglobin was 12.8 g/dL at D0 and 12.6 g/dL at DR. The geometric mean parasitaemia was 2553.5 parasites/mm^3^ at D0 and 4672.1 parasites/mm^3^ at DR (Table 2). The mean blood levels of CQ plus DCQ were higher than 100 ng/mL in all patients with recurrent infection. Thirty-five patients with CQ-susceptible *P. vivax* were randomly selected and included as controls (Table 2). Of the patients with CQ-susceptible *P. vivax*, 77.2% were males, mean age of 33.1 years. The mean haemoglobin was 13.2 g/dL. The geometric mean parasitaemia was 2623.9/mm^3^.

**Table 1:** Demographic and clinical characteristics of the study CQRPv admitted to a tertiary health centre, Manaus, Amazon, Brazil

Code	Sex/age	DR	Haemoglobin level in D0	Haemoglobin level in DR	Asexual malarial parasites/µL in D0	Asexual malarial parasites/µL in DR
R1	M/38	D28	12.7	14.1	1013.8	660.0
R2	M/1	D23	11.1	8.9	1000.0	8281.2
R3	M/40	D31	16.4	16.2	6901.0	281.4
R4	M/2	D28	10.7		5776.0	10,105.8
R5	M/35	D34	14.7	14.5	2105.6	2853.60
R6	M/8	D31	9.4	10.0	1136.8	4968.0
R7	M/46	D31	12.9	12.0	5059.2	4160.0
R8	F/21	D29	13.9	12.1	116.0	614.2
R9	F/21	D28	12.4	12.3	13,691.7	180.0
R10	F/43	D34	13.6	13.1	676.8	2613.2
R11	M/48	D30	14.9	14.7	21,285.0	10.0
R12	M/54	D27	14.0	15.1	343.0	154.8
R13	F/8	D32	10.2	9.5	690.0	2278.0
R14	M/22	D34	13.4	12.5	4347.0	6871.0
R15	M/41	D36	14.2	13.5	686.0	436.8
R16	M/41	D34	14.4	12.8	105.8	4507.7
R17	M/13	D38	12.4	12.0	2582.1	2613.2
R18	M/16	D32	13.5	12.9	78.0	11,096.2
R19	M/44	D31	12.2	14.0	8272.0	3162.0
R20	M/26	D25	15.1	15.6	1974.7	5385.6
R21	M/38	D28	14.0	14.4	2548.0	150.0
R22	M/1	D22		10.2	1380.0	21,360.0
R23	M/05	D28	11.4	10.0	4560.0	22,440.0
R24	M/5	D27	9.4	11.8	3120.0	1380.0
R25	F/21	D26	11.3	11.6	990.0	240.0

*D0* day admission, *DR* day of recrudescence, *M* male, *F* female

**Table 2:** Clinical characteristics of the study participants CQRPv and susceptible-chloroquine *P. vivax* admitted to a tertiary health centre, Manaus, Amazon, Brazil

	Resistant D0 (%)	IC 95%	Resistant DR (%)	IC 95%	Susceptible (%)	IC 95%
Gender						
Male	20 (80.0)	–	–	–	27 (77.2)	–
Female	5 (20.0)	–	–	–	8 (22.8)	–
Age (mean)	24.6	17.8–32.0	–	–	33.1	28.5–37.6
Asexual malaria parasites/µL	2553.5	1343.2–3763.8	4672.1	2214.0–7130.22	2623.9	1993.0–3254.7
Haemoglobin (mean)	12.8	12.1–13.6	12.6	11.9–13.4	13.2	12.7–13.7
Chloroquine concentrations (ng/mL)	–	–	230.1	182.8–276.1	–	–

D0 day admission, *DR* day of recrudescence

Asexual parasitaemia mean was similar at D0, D1, D2 and D3 in patients with CQRPv and CQ-susceptible *P. vivax* (P > 0.05). At D1, parasitaemia cleared in 2.9 and 0% of the patients with CQRPv and CQ-susceptible *P. vivax*, respectively (P = 0.659); 47 and 59.1% of the patients with CQRPv and CQ-susceptible *P. vivax* cleared at D2, respectively (P = 0.274); and 85.3 and 81.8% of the patients with CQRPv and CQ-susceptible *P. vivax* cleared at D3, respectively (P = 0.225).

For the microsatellite analysis at MS1F3, MS2 and MS8 loci, reading of the alleles was successful in 21 of the 25 CQRPv samples analysed. In these samples, 86.0% presented at least one concordant allele in primary infection and recrudescence (Additional file 4). The expected heterozygosity (H_E_) for *msp1*F3 was: 0.880 at D0 and 0.780 at DR; for MS2, 0.960 at D0 and  0.940 at DR; and for MS8 was 1.00 at D0 and 0.970 at DR.

### Analysis of copy number variation of the *pvcrt*-*o* and *pvmdr1* genes

**Fig. 1:**
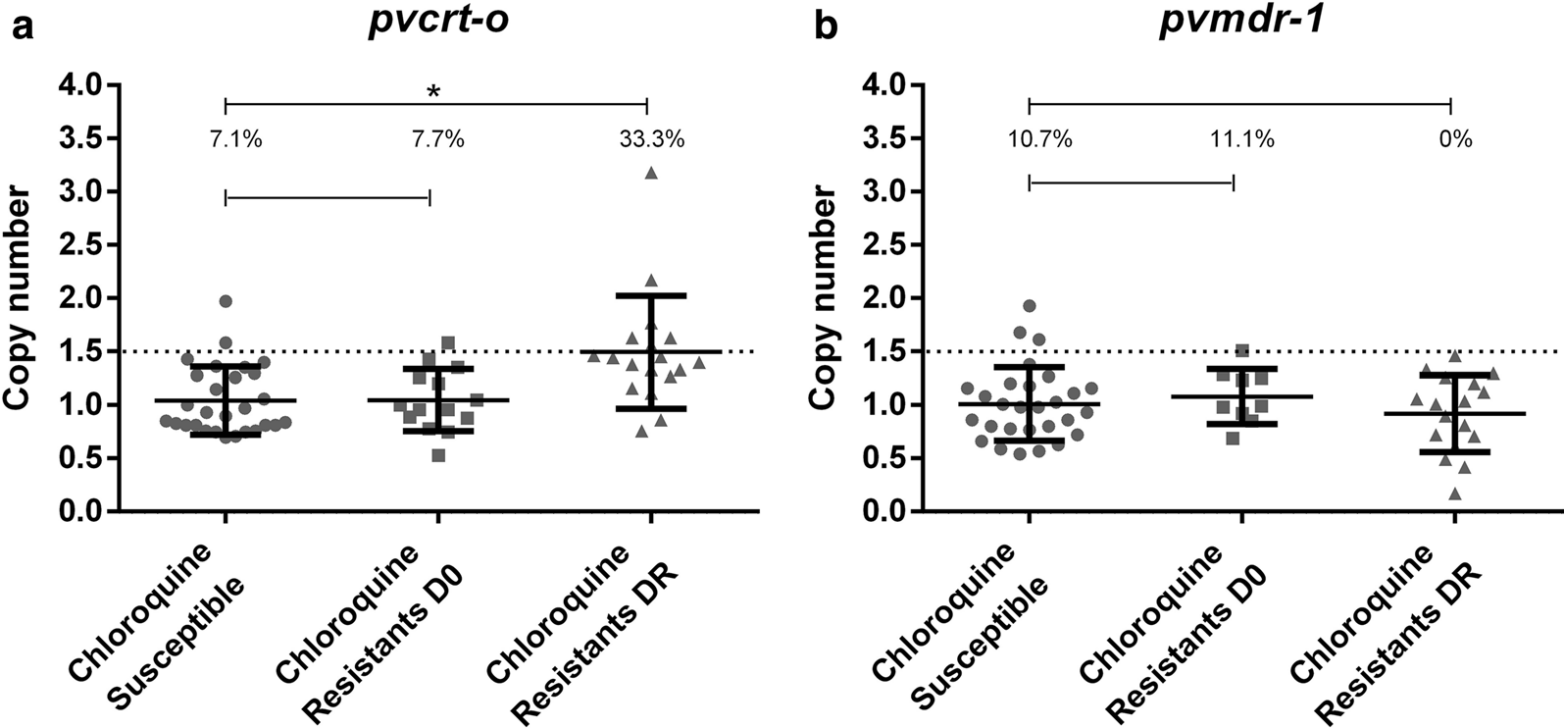
Determination of the copy number variation of genes in CQ-susceptible and patients resistant. **a** Determination of the number of copies for the *pvcrt*-*o* gene between the susceptible and resistant groups in D0 and DR. **b** Determination of the number of copies of the *pvmdr*-*1* gene between the susceptible and resistant groups in D0 and DR. *P < 0.05

For *pvcrt*-*o* CNV analysis, with 28 patients with CQ-susceptible *P. vivax* and 25 patients with CQRPv being analysed (14 samples from D0 to 18 from DR). At D0, 7.1% of the CQ-susceptible *P. vivax* presented multiple copies of *pvcrt*-*o*, 7.7% of the CQRPv showed multiple copies of *pvcrt*-*o* at D0 while at DR 33.0% had multiple copies of *pvcrt*-*o* (Fig. 1a). There was a significant difference between CQ-susceptible *P. vivax* and CQRPv at DR (P = 0.02).

For *pvmdr*-*1* CNV analysis, a total of 28 CQ-susceptible and 25 CQRPv (9 from D0 to 17 from DR) *P. vivax* samples were analysed. It was verified that 10.7% of the CQ-susceptible *P. vivax* samples, 11.1% of the CQRPv samples at D0 and 0.0% of the CQRPv samples at DR presented multiple copies (Fig. 1b). There was no significant difference between CQ-susceptible and CQRPv groups regarding *pvmdr*-*1* CNV (P > 0.05). Samples that did not meet the criteria used in the methodology were excluded from the analysis of these genes. Overall, *pvmdr1* and *pvcrt*-*o* CNV was not related to parasitaemia and age for individuals infected with *P. vivax* with either with multiple copies of *pvmdr*-*1* or with one copy of the genes (P > 0.05).

### Analysis of *pvcrt*-*o* and *pvmdr1* genes polymorphisms in the promoter region

**Fig. 2:**
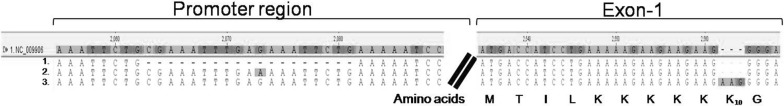
Scheme of the *pvcrt*-*o* gene showing promoter region and exon 1. This figure show specimens with 19 kb deletion without K10 insertion (1), samples with 19 kb insertion without k10 insertion (2) and samples with 19 kb insert with k10 insertion (3)

Nucleotide sequencing of a region flanking the promoter and exon I of the gene *pvcrt*-*o* showed a deletion of 19 bp in the promoter region at position 32,083–32,102 and an insertion of three bases in exon 1 leading to 5AAG repeats (AAG) after codon nine, leading to the K10 insertion compared to the reference sequence NC_009906-Chloroquine resistance transporter-*P. vivax* Sal1, with 4AAG repeats termed wild type here (Fig. 2).

A deletion of 19 bp was found in 11/23 (47.6%) of the patients with CQ-susceptible *P. vivax* and 3/10 (23.1%) of the samples with in CQRPv at D0. At day DR, 55.5% of the samples with CQRPv had the 19 bp deletion. There was no significant difference between groups (P ≥ 0.05, Fisher’s exact test) (Table 3).

All the samples with 19 bp deletion presented 4AAG repeats in the exon I (Table 2). Of the samples with wild type sequence, 8 (33.3%), 8 (60.0%) and 3 (25.0%) exhibited 5AAG (K10) repeats in the exon I of the gene in the samples with CQ-susceptible *P. vivax*, CQRPv at D0 and CQRPv at DR, respectively (Table 3).

For the *pvmdr*-*1 gene,* nucleotide sequencing was performed in 66 samples. Sixteen samples with CQ susceptible *P. vivax* and 23 CQRPv were sequenced, 14 in the D0 and 9 in the DR. There was no variation in the analysed gene compared to the *P. vivax* reference Sal-1 available in the NCBI gene bank.

## Discussion

Chloroquine and PQ are still the drugs of choice for the treatment of *P. vivax* in some countries, especially in Latin America [28–31]. However, CQRPv has emerged in several parts of the world [32, 33], including Malaysia [30], Myanmar [34], India [33] and Brazil [7, 35]. The molecular mechanism of CQRPv is still not well defined. In this study, CNV of *pvcrt*-*o* and *pvmdr1* was determined in patients with CQRPv from a well-characterized cohort under supervised treatment and this demonstrated a relationship between CQRPv in vivo and increased CNV of the *pvcrt*-*o*. These patients presented an effective concentration of CQ and DCQ and microsatellite revealed the presence of the same clonal nature at D0 and DR, indicating recrudescence. In Brazil, for the first time, CNV of the *pvcrt*-*o* was evaluated and the amplification of this gene was observed in *P. vivax* isolates in a study with treatment supervised for 42 days (Table 3).

**Table 3:** Frequency of the mutation and repetition AAG in the promoter region of the pv*crt*-*o* gene in CQRPv and CQ susceptible *P. vivax*

	Susceptible (%)	Resistant in D0 (%)	p	Resistant in DR (%)	p
Deletion 19 pb					
Yes	11 (47.6)	3 (23.1)	0.152	6 (55.5)	0.690
No	12 (52.4)	10 (76.9)		5 (44.5)	
Deletion 19 pb (4AAG)					
Yes	11 (47.6)	3 (23.1)	0.152	6 (55.5)	0.690
No	12 (52.4)	10 (76.9)		5 (44.5)	
Wild type (4AAG)					
Yes	2 (11.1)	1 (10.0)	0.927	1 (12.5)	0.919
No	21 (88.9)	11 (90.0)		10 (87.5)	
Wild type (5AAG)					
Yes	8 (33.3)	8 (60.0)	0.172	3 (25.0)	0.671
No	15 (66.7)	5 (40.0)		8 (75.0)	

In regions where mefloquine (MQ) is used to treatment of *P. falciparum* infection, drug pressure mediated by increase copy number variation of *pvmdr1* may select MQ-resistant *P. vivax* [36]. Actually, some studies show that *pvmdr1* amplification was associated to MQ-resistance [13, 23, 37, 38]. MQ was used in the Brazilian Amazon in the early 2000s, possibly contributing to the selection of this genotype in this region [5].

In this study, *pvmdr*-*1* CNV was not associated to CQRPv. Although CQ was found to be effective in India [39, 40], amplification of the *pvmdr*-*1* gene was found in 31.6% of the Indian isolates [33]. In Brazil, two studies described *pvmdr1* copy number variation, with an amplification rate of 0.9% in isolates from Acre state [41] and 20% in isolates from the states of Mato Grosso and Rondônia [22]. However, these studies did not have clinical follow-up to confirm CQRPv. *Pvmdr1* Y976F mutation was found in malaria-endemic regions where CQ is used as the first-line treatment [13, 38, 42].

In previous investigations, CQRPv isolates have shown high expression of *pvcrt*-*o* and/or *pvmdr*-*1* genes in a malaria reference centre in the Brazilian Amazon [11]. The main factor involved in gene expression regulation is its promoter, since it contains the binding sites for the RNA polymerases responsible for gene transcription [43]. To better elucidate CQRPv, the presence of mutations in the promoter region of the *pvcrt*-*o* and *pvmdr1* genes in CQ-susceptible *P. vivax* and CQRPv was investigated. For the *pvcrt*-*o* gene, an deletion 19 bp sequence was found in the promoter sequence, followed by 4AAG in the exon I. Although there was no significant difference in the frequency of mutations between these two groups, the CQRPv presented higher frequency of the deletion of 19 bp at D0 compared to at DR.

Here, we also described the occurrence of the wild type sequence in *pvcrt*-*o*, followed by 5AAG repeat (K10 insert) in CQ-susceptible *P. vivax* and CQRPv, with a frequency ranging from 25 to 60%. Similar frequency of insertion was reported by in vitro susceptibility to CQ studies using isolates from Thailand (46%) and Myanmar (56%) [13]. More recent studies have also demonstrated K10 insertion in isolates of *P. vivax* from India [32, 33] and Ethiopia [44], but the frequency was lower than that found in our study. Among the samples from India, a high frequency of polymorphisms in the *pvcrt*-*o* and *pvmdr1* genes has been demonstrated also, indicating the circulation of strains that may acquire CQRPv phenotype in that region [32, 33].

A limitation of this study is the small number of samples, restricting the possibility of detecting differences. Polyclonal infections were present, challenging the discrimination of recrudescence from relapse to re-infection. However, it was shown that plasma levels of CQ plus DCQ were higher than the minimum effective limits at DR for all patients. Results from in vivo studies, as the presented in this study, are very useful since they provide the response to treatment in the real life, but are subject to host immune influence, drug metabolism variations and drug quality [42]. Ex vivo assays evaluate drug activity in the absence of host confounding factors [45]. Then, potential influence of host immunity can not be discarded here.

## Conclusions

This was the first study with 42-day clinical follow-up to evaluate the variation of the number of copies and polymorphisms in the promoter region of the *pvcrt*-*o* and *pvmdr1* genes in relation to treatment outcomes. Significantly higher frequency of multi-copy *pvcrt*-*o* was found in CQRPv samples at DR compared to CQ-susceptible, indicating parasite selection of this genotype after CQ treatment and its association with CQ-resistance in vivo.

## Additional files

**Additional file 1.** Oligonucleotide primers used for copy number variation of *P. vivax* orthologs genes.

**Additional file 2.** Oligonucleotide primers used for promotor region sequencing of *P. vivax* orthologs genes.

**Additional file 3.** Oligonucleotide primers used for genotyping of *P. vivax.*

**Additional file 4.** Infection haplotypes in patients with CQRPv.
