# Supplementary material for: Chloroquine resistance is associated to multi-copy pvcrt-o gene in Plasmodium vivax malaria in the Brazilian Amazon
Source: Malar J. 2018 Jul 16;17:267. doi: 10.1186/s12936-018-2411-5 (PMC6048775; doi:10.1186/s12936-018-2411-5)
Supplement: Supplementary file 1 — Additional file 1. Oligonucleotide primers used for copy number variation of P. vivax orthologs genes. [file 12936_2018_2411_MOESM1_ESM.docx]

Additional file 1. Oligonucleotide primers used for copy number variation of *P.vivax* orthologs genes.

| **Gene** |  | **Sequence 5’ 🡪 3’** | **Use** |
| --- | --- | --- | --- |
| *pvcrt-o* | *pvcrt*F | 5'-TTTGGTCGCCGGGTCAT | Copy number variation |
|  | *pvcrt*R | 5'-CAGCAGCGAGATTAGCAAAAATT |  |
|  | *pvcrt*PROBE | FAM 5'-TGTTTAACCTCGTGTTGATTGCCTCGC |  |
| *pvmdr-1* | *pvmdr1*F | 5'-CAGCCTGAAAGATTTAGAAGCCTT | Copy number variation |
|  | *pvmdr1*R | 5'-CGGCTGTTGGAATCACTTTGA |  |
|  | *pvmdr1*PROBE | FAM 5'-CGGAGGAGTCGAACGAAGATGGTTTTTCTT |  |
| pvtubulin | pvtubulinF | 5'-TCGCTTAACGACGTCCCC | Copy number variation |
|  | pvtubulinR | 5'-TGGAATGTCACAAACGCTGG |  |
|  | pvtubulin probe | VIC 5'-TTCCGCTTCCCCCTCCACAGG |  |
