# Supplementary material for: Chloroquine resistance is associated to multi-copy pvcrt-o gene in Plasmodium vivax malaria in the Brazilian Amazon
Source: Malar J. 2018 Jul 16;17:267. doi: 10.1186/s12936-018-2411-5 (PMC6048775; doi:10.1186/s12936-018-2411-5)
Supplement: Supplementary file 2 — Additional file 2. Oligonucleotide primers used for promotor region sequencing of P. vivax orthologs genes. [file 12936_2018_2411_MOESM2_ESM.docx]

Additional file 2. Oligonucleotide primers used for promotor region sequencing of *P.vivax* orthologs genes.

| **Gene** | **Chromosome** | **Sequence 5’ 🡪 3’** | **Base pairs** | **Use** |
| --- | --- | --- | --- | --- |
| *pvcrt-o* | 1 | TCA ACC CGA ATC CAA ACC AGT G | 698 bps | Sequencing |
|  |  | GGC CTG CCT TAC TCT CAT TCT G |  |  |
| *pvmdr-1* | 10 | T ACT GCT GTT GCT ATT GTC CCT GGG | 1.002 bps | Sequencing |
|  |  | GAA TAT ATC ATT ATA CAG TGG |  |  |
