# Supplementary material for: Chloroquine resistance is associated to multi-copy pvcrt-o gene in Plasmodium vivax malaria in the Brazilian Amazon
Source: Malar J. 2018 Jul 16;17:267. doi: 10.1186/s12936-018-2411-5 (PMC6048775; doi:10.1186/s12936-018-2411-5)
Supplement: Supplementary file 3 — Additional file 3. Oligonucleotide primers used for genotyping of P. vivax. [file 12936_2018_2411_MOESM3_ESM.docx]

Additional file 3. Oligonucleotide primers used for genotyping of *P.vivax*.

| **Marker** | **Markertype** | **Primer name** | **Sequence 5' - 3'** | **Fluorescent dye** |
| --- | --- | --- | --- | --- |
| MS2 | Microsatellite | Primary forward | AGCACGACCAACAAGAGAGG | 6-FAM |
|  |  | Nested forward | GAGCTAGCCAAAGGTTCAAA |  |
|  |  | Nested reverse | TGGGGAGAGACTCCCTTTTC |  |
| MSP1F3 | Surface antigen | Primary forward | GGAGAACATAAGCTACCTGTCC | VIC |
|  |  | primary reverse | GTTGTTACTTGGTCTTCCTCCC |  |
|  |  | Nested forward | CAAGCCTACCAAGAATTGATCCCCAA |  |
|  |  | nested reverse | ATTACTTTGTCGTAGTCCTCGGCGTAGTCC |  |
| MS8 | Microsatellite | Primary forward | AAACGTAAAACCTTTGGCGG | 6-FAM |
|  |  | Nested forward | AGAGGAGGCAGAAATGCAGA |  |
|  |  | Nested reverse | CTGTCTTAGCCCCTTTGCGTTCTTTAT |  |
