# Supplementary material for: Chloroquine resistance is associated to multi-copy pvcrt-o gene in Plasmodium vivax malaria in the Brazilian Amazon
Source: Malar J. 2018 Jul 16;17:267. doi: 10.1186/s12936-018-2411-5 (PMC6048775; doi:10.1186/s12936-018-2411-5)
Supplement: Supplementary file 4 — Additional file 4. Infection haplotypes in patients with CQRPv. [file 12936_2018_2411_MOESM4_ESM.docx]

Additional File 4- Infection haplotypes in patients with CQRPv.

| **SAMPLE** | **MSP1F3** | | | | **MS2** | | | | | **MS8** | | | |
| --- | --- | --- | --- | --- | --- | --- | --- | --- | --- | --- | --- | --- | --- |
|  | Allele | Allele | Allele | Allele | Allele | Allele | Allele | Allele | Allele | Allele | Allele | Allele | Allele |
| R1 | 273 |  |  |  | 217 |  |  |  |  | 234 | 237 |  |  |
| R1_DR | 272 | 273 |  |  | 217 |  |  |  |  | 234 | 237 |  |  |
| R2 | 231 | 233 |  |  | 193 |  |  |  |  | 288 | 291 | 293 |  |
| R2_DR | 231 | 233 |  |  | 193 |  |  |  |  | Failure | | | |
| R3 | 232 |  |  |  | 192 | 194 |  |  |  | 237 | 291 | 293 |  |
| R3_DR | 232 |  |  |  | 192 | 194 | 217 |  |  | 237 | 291 | 293 |  |
| R4 | 232 |  |  |  | 194 |  |  |  |  | 222 | 225 |  |  |
| R4_DR | 232 |  |  |  | 192 |  |  |  |  | 222 | 225 |  |  |
| R5 | 231 | 233 | 273 |  | 193.0 |  |  |  |  | Failure | | | |
| R5_DR | 231 | 233 |  |  | 212 |  |  |  |  | Failure | | | |
| R6 | 231 | 233 |  |  | 193 | 212 |  |  |  | 257 | 260 | 291 | 293 |
| R6_DR |  | 233 |  |  | 193 |  |  |  |  | Failure | | | |
| R7 | Failure |  |  |  | Failure |  |  |  |  | Failure | | | |
| R7_DR | Failure |  |  |  | Failure |  |  |  |  | Failure | | | |
| R8 | 272 |  |  |  | 225 |  |  |  |  | 219 |  |  |  |
| R8_DR | 272 |  |  |  | 225 |  |  |  |  | 219 |  |  |  |
| R9 | 230 |  |  |  | 197 |  |  |  |  | Failure | | | |
| R9_DR | 230 |  |  |  | 197 |  |  |  |  | Failure | | | |
| R10 | 230 |  |  |  | 213 |  |  |  |  | 257 | 260 |  |  |
| R10_DR | 230 |  |  |  | 214 |  |  |  |  | 257 | 260 |  |  |
| R11 | 272 |  |  |  | 197 | 257 | 260 | 262 |  | Failure | | | |
| R11_DR | 272 |  |  |  |  | 222 | 225 | 231 | 262 | Failure |  |  |  |
| R12 | - | 230 | 272 | - | 213 | 269 | 276 |  |  | Failure | | | |
| R12_DR |  | 230 |  |  |  |  | 276 |  |  | 225 | 260 |  |  |
| R13 | 230 |  |  |  | 209 |  |  |  |  | 293 | 296 | 299 |  |
| R13_DR | 230 |  |  |  | 209 |  |  |  |  | 239 | 296 | 299 |  |
| R14 | 230 |  |  |  | 193 |  |  |  |  | 288 | 291 | 293 |  |
| R14_DR | 230 |  |  |  | 193 |  |  |  |  | 222 | 225 |  |  |
| R15 | 230 |  |  |  | 198 |  |  |  |  | 222 | 225 |  |  |
| R15_DR | 230 |  |  |  | 197 |  |  |  |  | Failure | | | |
| R16 | 231 | 280 |  |  | 213 |  |  |  |  | 234 | 237 |  |  |
| R16_DR | 232 | 280 |  |  | 213 |  |  |  |  | 234 | 237 |  |  |
| R17 | 298 |  |  |  | 198 |  |  |  |  | 219 |  |  |  |
| R17_DR | 298 |  |  |  | 197 |  |  |  |  | 234 | 237 |  |  |
| R18 | 273 |  |  |  | 197 |  |  |  |  | Failure | | | |
| R18_DR | 273 |  |  |  | 197 |  |  |  |  | 234 | 237 |  |  |
| R19 | 230 |  |  |  | 194 |  |  |  |  | Failure | | | |
| R19_DR | 230 |  |  |  | 193 |  |  |  |  | 294 | 296 |  |  |
| R20 | 232 |  |  |  | 201 |  |  |  |  | 285 | 288 |  |  |
| R20_DR | 232 |  |  |  | 201 |  |  |  |  | failure | | | |
| R21 |  |  | 264 |  | 209 |  |  |  |  | 254 | 257 |  |  |
| R21_DR |  |  |  |  |  |  |  |  |  | 254 | 257 |  |  |
| R22 | failure | | | | failure | | | | | failure | | | |
| R22_DR |  |  |  |  |  |  |  |  |  |  |  |  |  |
| R23 | failure | | | | failure | | | | | failure | | | |
| R23_DR |  |  |  |  |  |  |  |  |  |  |  |  |  |
| R24 | failure | | | | failure | | | | | failure | | | |
| R24_DR |  |  |  |  |  |  |  |  |  |  |  |  |  |
| R25 | failure | | | | failure | | | | | failure | | | |
| R25_DR |  |  |  |  |  |  |  |  |  |  |  |  |  |
